# Effective Identification of Low-Gliadin Wheat Lines by Near Infrared Spectroscopy (NIRS): Implications for the Development and Analysis of Foodstuffs Suitable for Celiac Patients

**DOI:** 10.1371/journal.pone.0152292

**Published:** 2016-03-28

**Authors:** María Dolores García-Molina, Juan García-Olmo, Francisco Barro

**Affiliations:** 1 Departamento de Mejora Genética, Instituto de Agricultura Sostenible (IAS), Consejo Superior de Investigaciones Científicas (CSIC), Córdoba, Spain; 2 NIR/MIR Spectroscopy Unit, Central Service for Research Support, University of Córdoba, Córdoba, Spain; Murdoch University, AUSTRALIA

## Abstract

**Scope:**

The aim of this work was to assess the ability of Near Infrared Spectroscopy (NIRS) to distinguish wheat lines with low gliadin content, obtained by RNA interference (RNAi), from non-transgenic wheat lines. The discriminant analysis was performed using both whole grain and flour. The transgenic sample set included 409 samples for whole grain sorting and 414 samples for flour experiments, while the non-transgenic set consisted of 126 and 156 samples for whole grain and flour, respectively.

**Methods and Results:**

Samples were scanned using a Foss-NIR Systems 6500 System II instrument. Discrimination models were developed using the entire spectral range (400–2500 nm) and ranges of 400–780 nm, 800–1098 nm and 1100–2500 nm, followed by analysis of means of partial least square (PLS). Two external validations were made, using samples from the years 2013 and 2014 and a minimum of 99% of the flour samples and 96% of the whole grain samples were classified correctly.

**Conclusions:**

The results demonstrate the ability of NIRS to successfully discriminate between wheat samples with low-gliadin content and wild types. These findings are important for the development and analysis of foodstuff for celiac disease (CD) patients to achieve better dietary composition and a reduction in disease incidence.

## Introduction

Wheat is the most widely cultivated cereal in the world due to its adaptability to different environments and high yields, as well as to the unique biomechanical properties of wheat dough. Although in wheat grain proteins are in minor proportion (9–15%) compared to starch (60–75%), they are essential for wheat functionality. With respect to dough functionality, two major groups of proteins are recognised in wheat grains: gluten proteins, responsible for bread-making quality; and non-gluten proteins, with mainly structural function and a secondary role in bread-making. Gluten proteins account for between 80–85% of total grain protein, and include the majority of storage proteins, with about 30% being gliadins and 50% glutenins [1]. Gluten proteins are referred to as prolamins due to their high content of proline and glutamine [2, 3]. The gliadins are monomeric and they are classified into three main families: α/β-, ω- and γ-gliadins, whereas the glutenins are present as polymeric complexes, classified into two groups; the high molecular weight (HMW) and low molecular weight (LMW) subunits of glutenin. The two protein types have different functional properties; gliadins are responsible for extensibility and viscosity of doughs, while the glutenins contribute elasticity and form large polymers linked by disulphide bonds.

In addition to their role in dough functionality, gluten proteins are also associated with two important human enteropathies, which appear to be increasing in importance and may affect up to 7% of the population: i) celiac disease (CD) [4], which affects both children and adults at a frequency of about 1% worldwide, and ii) non-celiac gluten sensitivity (NCGS) [5] with an estimated prevalence of 6% in the USA population. CD is the most studied of these pathologies, it is a food sensitivity enteropathy caused by the ingestion and exposure to gluten not only from wheat, but also from barley, and rye [6]. CD affects about 1% of the population in Western countries [7], but it is thought to be an under-diagnosed disease. CD is triggered by exposure to gluten and has a genetic component, with the genes encoding the human leukocyte antigen (HLA) DQ2 or DQ8 molecules present in 95% and 5%, of CD patients respectively, conferring a higher risk of disease [8]. Isolation and characterization of T cells from CD patients reveals different but similar DQ2 and DQ8 epitopes. Such epitopes are generally rich in proline and glutamine residues, and most of them reside in the gliadin fraction of gluten [9, 10].

People suffering either pathology are recommended a gluten-free diet (GFD) for life. Clinical manifestations and symptoms associated with CD ameliorate with a GFD. However, a full GFD is complicated to follow, as wheat and its derivatives constitute an important food additive widely used by industry, and therefore gluten proteins may be found in many unexpected food types such as meat, fish, milk, and many other foodstuffs. Moreover, the presence of immunogenic proteins in rye (secalins) and barley (hordeins) makes food products made with these cereals also unsuitable for CD patients. Transgressions, intentional or not, in GFDs are very common and may affect between 32 and 55% of CD patients [11]. At the same time, a full GFD may be detrimental to gut health as it can lead to reductions in beneficial gut bacteria microbiota [12].

The development of new cereal varieties, and specially wheats, with reduced immunogenic gluten content would be extremely significant for CD and NCGS sufferers, both to improve their diet and to reduce the incidence of disease, as it has been shown that disease initiation is associated with the amount and duration of gluten exposure [13]. In the case of wheat, recognition of the prevalence of intolerance by consumers has lead towards the perception of wheat as a “bad” foodstuff and a boom in gluten-free foods, although many of these have lower organoleptic quality than wheat, are typically highly-processed and may contain undesirable additives. Many of these consumers do not need to observe a GFD, but wish to reduce their gluten intake and there are clear social and economic drivers for the development of low-gluten cereal products with excellent organoleptic and nutritional properties and suitable for consumption by a broad group of people [14].

One promising approach for reducing gluten toxicity and, therefore, the incidence of gluten-related intolerances from cereals is the down-regulation of immunodominant gluten peptides by RNA interference (RNAi). This technology has been used to down-regulate the expression of particular gliadin fractions [15–17] and total gliadins [18] in bread wheat. Protein extracts from flour of lines with all gliadin fractions down-regulated were tested for their capacity to stimulate DQ2- and DQ8- restricted T-cell clones from CD patients, and a pronounced reduction in proliferative responses was seen for some transgenic lines [18]. Flours from these lines showed increased stability and better tolerance to over-mixing [19], and had improved nutritional properties since their lysine content was significantly higher than that of normal wheat flour [14]. Moreover, conservative estimates indicated that CD patients could safely consume about 67 grams of bread per day made with such low-gliadin flour, and this amount could be higher if flour was blended with other non-toxic cereals like rice, maize, or oat. Therefore, these down-regulated lines are excellent candidates as raw material for developing food products that may be tolerated by CD patients and people with NCGS, increasing the range of available food products and enhancing their diet and quality of life. A major advantage of these products is that their high digestibility, coupled with high palatability will make them suitable for all consumer groups, and not just those with known gluten intolerances.

To facilitate the use of these new wheats, a system capable of distinguishing between normal wheat lines and low-gliadin lines is required, enabling the rapid identification of samples that will be used for the development of products suitable for celiac patients. Near Infrared Spectroscopy (NIRS) is a method of analysis that uses the Near-Infrared Region of the electromagnetic spectrum (800-2500nm). NIRS is a non-destructive technique that requires small samples [20], generating instant results, which entails a reduction in time and costs. In contrast to ELISA assays, NIRS does not require technical expertise or complex techniques, the spectrophotometer can be installed anywhere with no requirement of reagents or complicated protocols.

Because of its simplicity, it is the method of choice for predicting and discriminating nutritionally important components in foods and, in particular, flours and grains [21]. NIRS has been used for screening acrylamide content in potato crisps [22] and for the determination of non-starch polysaccharides in cereal grains [23]. Cocchi et al. [24] applied NIR technology to determine the Synthetic Index of Quality in bread wheat flour, which is composed of parameters such as hectolitre weight, falling number, protein content, etc. NIR spectroscopy has also been used to discriminate between control and naturally contaminated wheat samples containing deoxynivalenol, a mycotoxin present in cereal grains and associated with *Fusarium spp*. infections. The model, correctly classified almost 70% of the validation samples, but misclassified samples showed deoxynivalenol contamination levels close to the cut-off level [25].

The objective of the present study was to develop a NIR-based technology for the effective identification of low-gliadin wheat samples generated by RNAi, from wheat lines containing the full set of gliadins. Moreover, results on the development of NIRS discrimination models usable in whole grain and flour samples are reported, as the analysis of whole grain is faster and more inexpensive than flour analysis.

## Materials and Methods

### Plant material

Low-gliadin transgenic lines of bread wheat used in this study were reported previously [18, 26], and have either individual or all three groups of gliadins down-regulated. These lines were obtained using the plasmid combinations indicated in S1 Table. Non-transgenic wheat lines from genotypes with full gliadin content were used as control lines. Thus, 46 and 13 transgenic lines from bread wheat (*Triticum aestivum*) cvs. Bobwhite, BW208 and BW2003, respectively, were used to carry out the calibration equation, while, for external validation, 46 lines from Bobwhite 208 and two lines from each of the varieties Anza, Arelo, Gazul, Panifor, T590, THA10, THA3, THA53, THA7, were used. The sets of transgenic and control lines were assayed during five years (2010 to 2014) as described previously [27], providing 1105 samples, of which 535 were grain and 570 flour. The total population of spectra was divided into training and validation sets, with 274 and 78 samples for whole grain, and 308 and 78 samples for flour, respectively. The validation set was from the year 2013. The second external validation, with samples from the year 2014, was performed using 183 samples for whole grain and 184 samples for flour.

After harvesting, low-gliadin and non-transgenic wheat lines were threshed and cleaned. Samples were then processed in two different sets for NIR analysis. One set was milled (Tecator Cyclotec 1093 Sample Mill, Foss SA, Sweden) with a maximum sample size of 1 mm. This set was used for NIRS discriminations in flour, and the other set, unmilled, was used for discrimination in whole grain.

Gliadins and glutenins were quantified by means of Reversed-Phase High Performance Liquid Chromatography (RP-HPLC) as described [28]. Three independent repetitions were carried out for each transgenic line and control.

The protein content of flour was calculated from the Kjeldahl nitrogen content (%N x 5.7) according to the standard ICC method no. 105/2 (ICC, 1994).

### Analysis of wheat flour by R5 competitive ELISA

Gluten content of samples from low-gliadin transgenic wheat lines and control lines containing the full set of gliadins were analysed at Centro Nacional de Biotecnología (CSIC, Campus of Cantoblanco, 28049-Madrid) using the R5 monoclonal antibody as described elsewhere [29]. The R5 assay is the “*Codex Alimentarius* Standard for Gluten-Free Foods” [30] (ALINORM08/31/26, 2008). The assay was performed in duplicate for each sample and results expressed in parts per million (ppm).

### Equipment and Collection of Spectra

Grain and flour samples were scanned using the Foss-NIRSystems 6500 System II (Foss-NIRSystems Inc., Silver Spring, MD, USA). This instrument is equipped with two autogain detectors: one for 400–1100 nm (known as the visible region (VIS region)) and another for 1100-2500nm (known as the NIR region).

The samples were placed in a rectangular quartz capsule with a window of 4.5x5.5 cm^2^. Each sample was analysed in duplicate to reduce measuring errors. Reflectance spectra were collected every 2 nm from 400 to 2500 nm. The absorbance data were obtained and stored as log (1/R), where R is the reflectance. The two spectra of the flour and the two spectra of the whole grain were averaged to obtain, respectively, the mean spectrum that was used to determine the classification between transgenic and non-transgenic wheat. WinISI II software version 1.50 (Infrasoft International Port Matilda, PA, USA) was used to process the data. All determinations were performed by the same operator.

### Discriminant Analysis

#### WinISI II analysis

The training set of transgenic and control wheat samples was analyzed using WinISI II version 1.50 software. Samples from the years 2010, 2011 and 2012 were used to develop discriminant models (Table 1A) whereas the samples collected in 2013 and 2014 were used for external validation and to corroborate the results obtained during the external validation, respectively (Table 1B). During the development of classifications, the process relates only spectral information and it is necessary to assess the discriminant capacity of the models, using an independent set of samples that have not been used during the classification process.

**Table 1:** Total protein content, gliadin and glutenin distribution, and gluten content determined by R5 monoclonal antibody assays of non-transgenic and transgenic lines from years 2010, 2011 and 2012, used for the development of classification models (A) and, from years 2013 and 2014, used for external validation (B).

**A**						
	**Non-transgenic**			**Transgenic**		
	**Average**	**Max**	**Min**	**Average**	**Max**	**Min**
**Protein (% DW)**	13.9	17	12.2	14.3	18	11.2
**Gliadin (μg/mg flour)**	54.6	98.9	11.5	28.3	76.4	8
**Glutenin (μg/mg flour)**	23.4	36.4	11.8	27	50.5	10.1
**R5 (ppm)**	122165	154362	89189	49077	181986	3057
**B**						
	**Non-transgenic**			**Transgenic**		
	**Average**	**Max**	**Min**	**Average**	**Max**	**Min**
**Protein (% DW)**	12.2	16.4	8.3	13	16.3	11.1
**Gliadin (μg/mg flour)**	50.5	74.6	16.4	19.6	31.3	10.6
**Glutenin (μg/mg flour)**	26.9	57.7	14.5	41.6	75	14.3
**R5 (ppm)**	82808	112970	57083	34468	128909	1736

Discriminant Partial Least Square (DPLS) analysis was employed to determine the origin of the samples. DPLS correlates spectral variations with the low-gliadin transgenic and control wheat samples, and maximizes the covariance between the two variables, in order to developing a classification model. Samples are classified according to the value of a dummy variable predicted by the DPLS model, which preferably should be close to the values used to codify the class. Low-gliadin transgenic wheat samples were assigned a value of 2 and control wheat lines containing the full set of gliadins a value of 1.

Scatter correction was carried out using the standard normal variate and a de-trending (SNV+DT) algorithm. Different ranges of the 400–2500 nm spectrum and different combinations of derivative mathematic treatments were applied to the spectral data. WinISI derivative mathematic treatments are denoted by a four letter (a, b, c, d) code where ‘a’ is the derivative degree, ‘b’ is the wavelength where this derivative is calculated, ‘c’ is the smoothing segment, and ‘d’ is the second smoothing segment. The use of derivatives reduces or removes the backgrounds of the spectral data, thus, when the first derivative is used, an overlap of spectral peaks occurs and the linear background comes to a constant level, while, with the second derivative the level of this background is brought to zero [21].

## Results and Discussion

Table 1 summarises the whole grain protein characteristics of the lines used in this study. As expected, the transgenic wheat lines have lower contents of gliadins than non-transgenic samples. However, the glutenin fraction of gluten was, in general, increased in transgenic samples, as well as the non-gluten protein fraction, so that the total protein contents of transgenic lines are comparable to that of non-transgenic samples. The gluten content of low-gliadin transgenic lines and non-transgenic lines was determined by ELISA assay, using the monoclonal antibody R5. The average from all non-transgenics used during calibration (Table 1A) and validation (Table 1B) is compared with the average from transgenic lines and a reduction in gluten content as measured by R5 assay can be observed in transgenic lines, with a wide range of variation among different lines, which were obtained using different plasmid combinations with different efficiency for the down-regulation of gliadins [26].

Although flour from low-gliadin transgenic wheat lines can be distinguished by protein extraction and A-PAGE profiles, the use of NIR technology has many advantages for industrial use such as speed and simplicity, it is non-destructive, and can easily be implemented for product traceability at any point in the food chain. One important question to be addressed is to define which is the best sample material (grain or flour) for qualitative analysis with NIRS. Table 2 shows sample distribution for the classification and validation sets. As shown, 71 samples from non-transgenic wheats, and 203 samples from transgenic lines were used for grain classification whereas 100 and 208 samples from non-transgenic and transgenic lines respectively, were used for flour classification experiments. The external validation using samples from the 2013 season was carried out using 78 samples, of which 9 were non-transgenic and 69 were transgenic lines (Table 2). In addition, a second external validation, to corroborate the results, was carried out, using both grain and flour samples from the year 2014 (Table 2).

**Table 2:** Numbers of wheat samples used in the training and validation sets for whole grain and flour.

	Year 2013				Year 2014			
	Whole grain		Flour		Whole grain		Flour	
Samples	Classification	Validation	Classification	Validation	Classification	Validation	Classification	Validation
**Non-transgenic**	71	0	100	0	71	0	100	0
**Transgenic**	203	0	208	0	203	0	208	0
**Non-transgenic**	0	9	0	9	0	46	0	47
**Transgenic**	0	69	0	69	0	137	0	137

### Develop of Models

To evaluate the influence of different wavelength regions, several classification models were performed using the full spectrum (400–2500 nm), the NIR region (800–1100 nm, 1100–2500 nm), and the visible region (400–780 nm). The models were then evaluated using two independent external validations employing the set of samples from the years 2013 (Table 3) and 2014 (Table 4), which were not used in the training step. Before developing the classification models, different pre-treatments and mathematical treatments were applied to the spectral data to eliminate baseline shifts, slope changes and curvilinearity of spectra caused by diversity of the particle size, scattering and other influencing factors [31]. To perform the validation of the qualitative analyses, the number of samples that were classified correctly and incorrectly was determined.

**Table 3:** Models for the classification and validation of wheat whole grain or flour samples; spectral range 400–2500 nm (A); spectral range 1100–2500 nm (B). Samples from year 2013 were used for external validation.

**A**						
	**No pre-treatment**		**Pre-treatment SNV+DT**			
	**Derivative (0, 0, 1, 1)**		**Derivative (1, 4, 4, 1)**		**Derivative (2, 10, 5, 1)**	
	**Whole grain**	**Flour**	**Whole grain**	**Flour**	**Whole grain**	**Flour**
***Classification***						
No. samples misclassified	4/274	3/308	0/274	1/308	0/274	1/308
Classification error (%)	1.5	1	0	0.3	0	0.3
***Validation***						
No. samples misclassified	9/78	8/78	6/78	4/78	6/78	9/78
Validation error (%)	11.5	10.3	7.7	5.1	7.7	11.5
**B**						
	**No pre-treatment**		**Pre-treatment SNV+DT**			
	**Derivative (0, 0, 1, 1)**		**Derivative (1, 4, 4, 1)**		**Derivative (2, 10, 5, 1)**	
	**Whole grain**	**Flour**	**Whole grain**	**Flour**	**Whole grain**	**Flour**
***Classification***						
No. samples misclassified	2/274	3/308	3/274	3/308	2/274	4/308
Classification error (%)	0.73	0.97	1.09	0.97	0.73	1.3
***Validation***						
No. samples misclassified	10/78	2/78	9/78	3/78	2/78	5/78
Validation error (%)	12.8	2.5	11.5	3.8	2.5	6.4

**Table 4:** Models for the classification and validation of wheat whole grain or flour samples; spectral range 400–2500 nm (A); spectral range 1100–2500 nm (B). Samples from year 2014 were used for external validation.

**A**						
	**No pre-treatment**		**Pre-treatment SNV+DT**			
	**Derivative (0, 0, 1, 1)**		**Derivative (1, 4, 4, 1)**		**Derivative (2, 10, 5, 1)**	
	**Whole grain**	**Flour**	**Whole grain**	**Flour**	**Whole grain**	**Flour**
***Classification***						
No. samples misclassified	4/274	3/308	0/274	1/308	0/274	1/308
Classification error (%)	1.5	1	0	0.3	0	0.3
***Validation***						
No. samples misclassified	13/183	5/184	8/183	2/184	7/183	15/184
Validation error (%)	7.1	2.7	4.4	1.1	3.8	8.1
**B**						
	**No pre-treatment**		**Pre-treatment SNV+DT**			
	**Derivative (0, 0, 1, 1)**		**Derivative (1, 4, 4, 1)**		**Derivative (2, 10, 5, 1)**	
	**Whole grain**	**Flour**	**Whole grain**	**Flour**	**Whole grain**	**Flour**
***Classification***						
No. samples misclassified	2/274	3/308	3/274	3/308	2/274	4/308
Classification error (%)	0.7	1	1.1	1	0.7	1.3
***Validation***						
No. samples misclassified	22/183	6/184	8/183	8/184	9/183	4/184
Validation error (%)	12	3.3	4.4	4.3	4.9	2.2

When all spectrum regions were used (400–2500 nm) and the validation was carried out using samples from the year 2013 (Table 3A), the classification and validation errors in the best model of whole grain were 0 and 7.7% respectively. In this case, the same results were obtained when the first and second derivatives were used with scatter correction (SNV+D), where six samples from a total of 78 samples were misclassified in the validation process but all non-transgenic were classified correctly using the first derivative. However, using the second derivative all whole grain transgenic samples were classified correctly while six controls were misclassified (Table 3A). Accordingly, the best model was obtained when the first derivative was used as the transgenic population is greater than the non-transgenic (69 transgenic vs. 9 non-transgenic lines), and therefore the error is reduced. Regarding the flour models, the best results were obtained when the first derivative with scatter correction was used (Table 3A). In this case the total classification error was 0.3%, and the external evaluation provided 5.1% of the validation error, as four samples (all non-transgenic) were classified incorrectly.

When only the NIR region (1100–2500 nm) was used and the validation was carried out using the same samples from year 2013 (Table 3B), the best classification error for whole grain was 0.7%. In this group of samples, the validation error was 2.5% when the scatter correction and second derivative was used. In the case of flour, we obtained a classification error of 1% and a validation error of 2.5% with no pre-treatment (no SNV+D). In both whole grain and flour, the number of misclassified samples was two, one transgenic and one non-transgenic.

**Fig 1:**
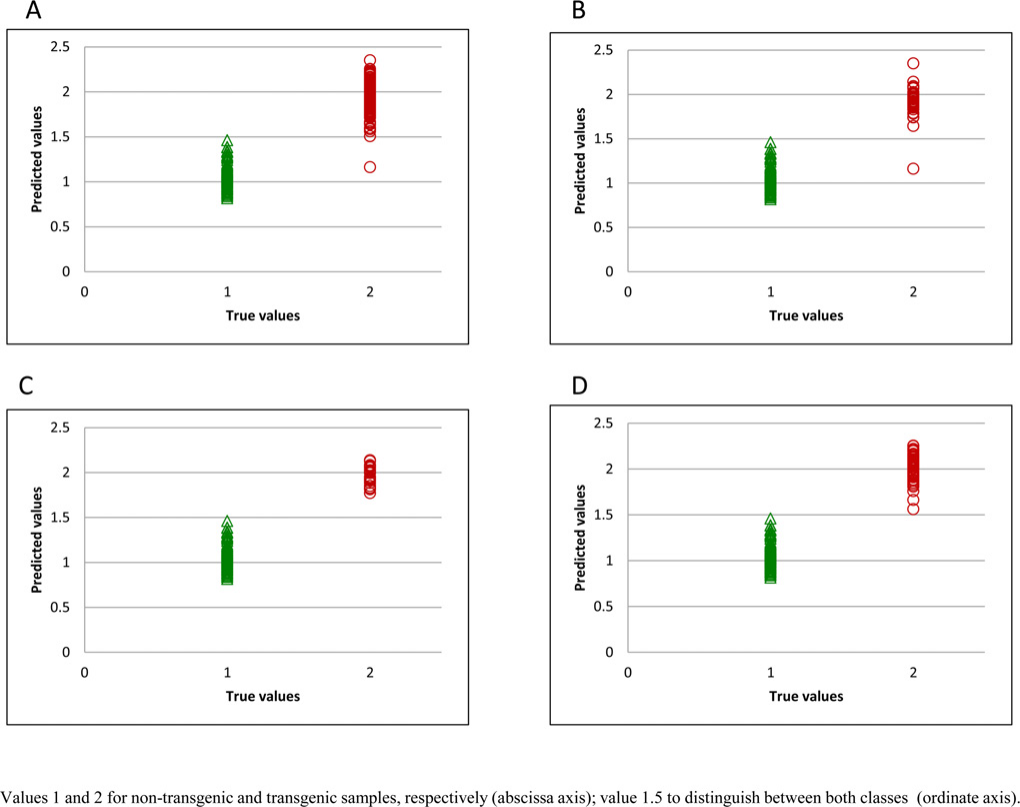
Predicted flour values for training set. All transgenic samples (A), and samples obtained using plasmid combinations 3 (B), 4 (C), and 5 (D).

A second external validation using samples from year 2014 (Table 4) was performed to verify the results obtained during the first validation, given that environmental conditions during 2013 were different from the other years; in particular the rainfall pattern, which strongly influences the availability of nutrients such as nitrogen, and, consequently, affects total protein content. To carry out this second validation, 184 samples were used. It was observed that the results were very similar to those found with samples from year 2013. Table 4A shows that the best validation model for whole grain was obtained using the second derivative with scatter correction (SNV+D). In this case, the validation error was 3.8%, as seven samples (five transgenic and two non-transgenic) were misclassified. However, for flour samples, the lowest percentage error (1.1%) was obtained with the first derivative and scatter correction (SNV+D), with only two transgenic samples being misclassified. When the spectral range 1100–2500 nm was used (Table 4B), the best results for validation were 4.4% using the first derivative and 2.2% with the second derivative, for whole grain and flour samples respectively. All values resulting from the classification and validation model, showed between 8 and 10 PLS discriminant factors.Thus the results obtained indicate that the best discrimination model came from using the full spectrum (400–2500 nm) for both whole grain and flour samples, particularly in the case of the flour, in which the validation error was 1.1%. Fig 1 shows the distribution of the spectral populations of flour samples for the best classification model (Fig 1A). Giving that the down-regulation of gliadins was not equally effective with each plasmid combination [18, 26], we present the spectral populations for each of the different plasmid combinations used (Fig 1B, 1C, 1D and S1 Fig). As reported previously, lines which presented a lower toxicity corresponded with plasmid combinations 4, 5, 6 and 7 where reductions of 84%, 93%, 88% and 92% in gluten content were measured by competitive monoclonal antibody assays. In a previous study, transgenic wheat lines from plasmid combinations 4, 5, 8 y 9 were used by Gil-Humanes et al. [14] to produce high quality breads with improved nutritional value. In that study, breads were made with flour of low-gliadin lines and non-transgenic wheat lines, and from rice, as in typical gluten-free breads. They concluded that wheat lines with very low gliadin content showed organoleptic properties comparable with normal breads and better than rice breads. These low-gliadin lines, which have good prospects for commercialization in low-gluten foods, were correctly classified using the NIR technology (Fig 1B, 1C and 1D and S1 Fig).

**Fig 2:**
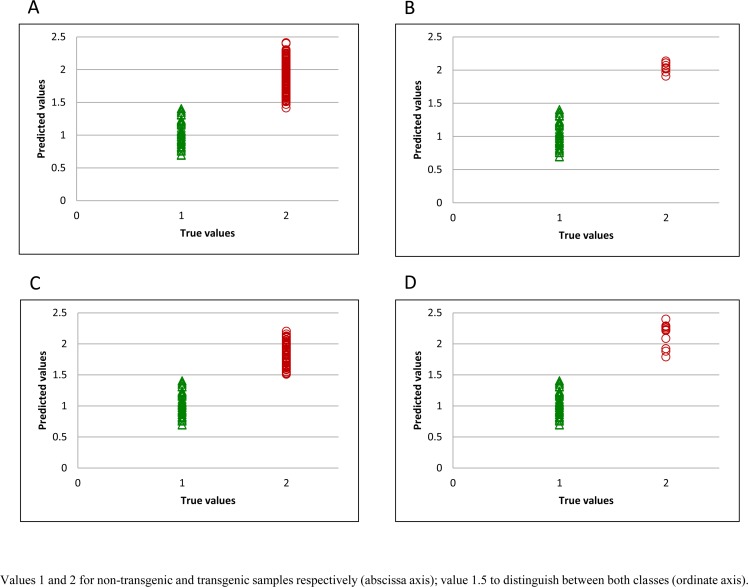
Predicted flour values for validation set. All transgenic samples (A), and samples obtained using plasmid combinations 3 (B), 4 (C), and 5 (D).

For developing the classification model, samples were classified using a binary code in a dummy variable, with transgenic samples having a value of 2 and non-transgenic samples a value of 1. Thus, the limit value for the simulated variable to distinguish between the two classes is 1.5. During the training step, the only sample misclassified was found in the spectral population from silencing plasmid combination 3 (Fig 1B). Fig 1C and 1D represent the plasmid combinations 4 and 5 respectively, previously described as best candidate samples for commercialization. It can be seen that all samples are correctly classified. The best distribution for external validation, with flour samples from year 2014 is represented in Fig 2A. The two transgenic samples which were misclassified belonged to plasmid combination 1 (S2A Fig) and plasmid combination 7 (S2D Fig).

The transgenic wheat lines used in this work were grown in different seasons and therefore subject to different environment conditions. It is likely that growth under different conditions, may affect discrimination [32] due to differences in water and nutrient availability and temperature regimes leading to variation in the grain composition in both transgenic and non-transgenic lines. Wieser and Seilmeier [33] have shown that the environment influences both protein composition and total protein.

In our case, total protein content did not differ between transgenic and non-transgenic lines for the same year, which may indicate that both transgenic and non-transgenic samples have similar grain nitrogen composition. However, there was strong year-to-year variation for both sets of samples. Consequently, the correct classification of the samples suggests that in the transgenic samples, containing a reduced content of gliadins, compensation occurs in other protein fractions. Gil-Humanes et al., [34] found several transgenic lines in which the suppression of gliadins was associated with compensatory increases in glutenin, albumin and globulin fractions. Thus, the ratio of glutenins to gliadins was higher than that for non-transgenic lines. Others authors who have analysed the protein profile in wheat lines exhibiting silencing of ω-5 gliadins [16] and α-gliadins [17] have also reported increases in non-gluten proteins. These results suggest the existence of a compensatory mechanism operating in response to the down-regulation of specific gluten protein groups. However, despite the compensation effect, the results reported here show that NIR can distinguish between transgenic (low-gliadin) and non-transgenic wheats.

Approximately 80% of the transgenic wheat samples misclassified correspond to those having the plasmid combination 1 (S1 Table). This may be because lines with this fragment combination do not show a reduction in the ω-, γ-gliadin fractions and LMW glutenin content [26], and therefore, their protein profile is more similar to the wild type and as a result, NIRS is not able to make a correct discrimination.

One goal of the study was to determinate the best material to perform the analysis, given that, diversity in particle size in the test material will affect scattering and is a major source of variation in NIR spectra [35]. Although the results obtained for whole grain were similar to those for flour, they tend to become more consistent when the sample is milled. For whole grain the best results were obtained using the second derivative and scatter correction (SNV+DT), probably because the grain size is more heterogeneous in the whole grain samples, and scatter correction plays a more important role than that in flour samples, where the second derivative was not necessary [36].

## Conclusions

The development of wheat varieties with reduced immunogenic prolamin fractions may contribute not only to improving the diet of CD patients and NCGS sufferers but may also be useful for anyone who decides to follow a gluten free diet. Moreover, the development of low-gluten cereal products will help improve organoleptic quality and, especially, the nutritional profile for people who follow a low gluten / GFD. In the commercialization of gluten-free foods, traceability is essential to ensure that gluten-free products do not suffer cross-contamination to ensure products suitable for consumption by CD or NCGS sufferers. We have shown that discrimination between transgenic low-gliadin wheats and non-transgenic wheat is possible using NIR technology, and we have developed a robust model for classification. Although assays on whole grain give advantages over flour assays because of savings in time and money, results in flour were more reliable than in whole grain.

## Supporting Information

S1 Fig Predicted flour values for training set. Samples obtained using plasmid combinations 1 (A), 2 (B), 6 (C), 7 (D), 8 (E) and 9 (F).
(TIF)

S2 Fig Predicted flour values for validation set. Samples obtained using plasmid combinations 1(A), 2 (B), 6 (C) and 7 (D).
(TIF)

S1 Table Plasmid combinations used: Combination number 0 indicates BW208 wild type wheat and numbers 1–9 show the plasmid combinations used to generate lines in years 2010, 2011, 2012, 2013 and 2014. (DOCX)
