# Supplementary figures and images for: Effective Identification of Low-Gliadin Wheat Lines by Near Infrared Spectroscopy (NIRS): Implications for the Development and Analysis of Foodstuffs Suitable for Celiac Patients
Source: PLoS One. 2016 Mar 28;11(3):e0152292. doi: 10.1371/journal.pone.0152292 (PMC4809495; doi:10.1371/journal.pone.0152292)

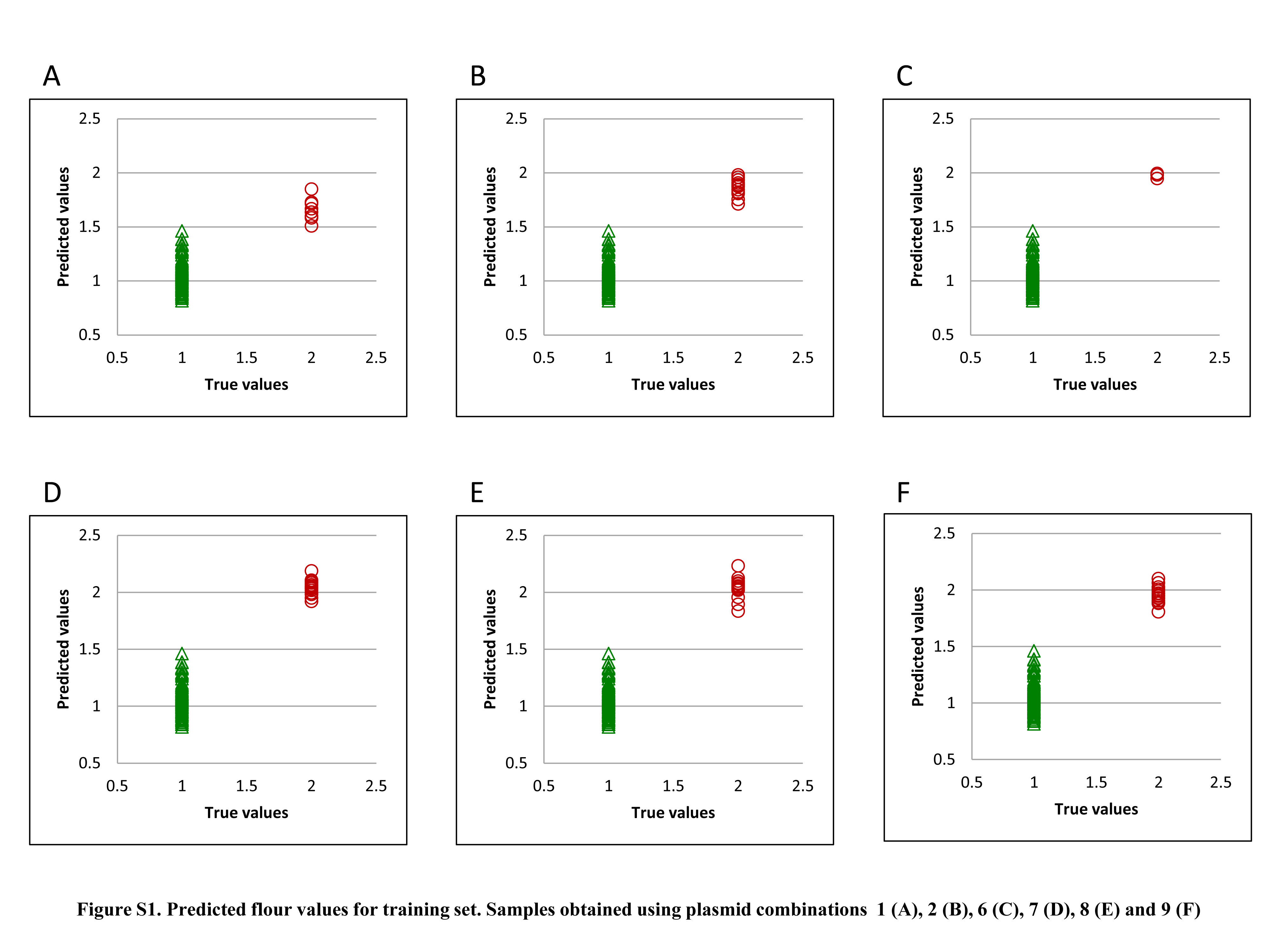

Supplement: S1 Fig — Samples obtained using plasmid combinations 1 (A), 2 (B), 6 (C), 7 (D), 8 (E) and 9 (F). (TIF) [file pone.0152292.s001.tif]

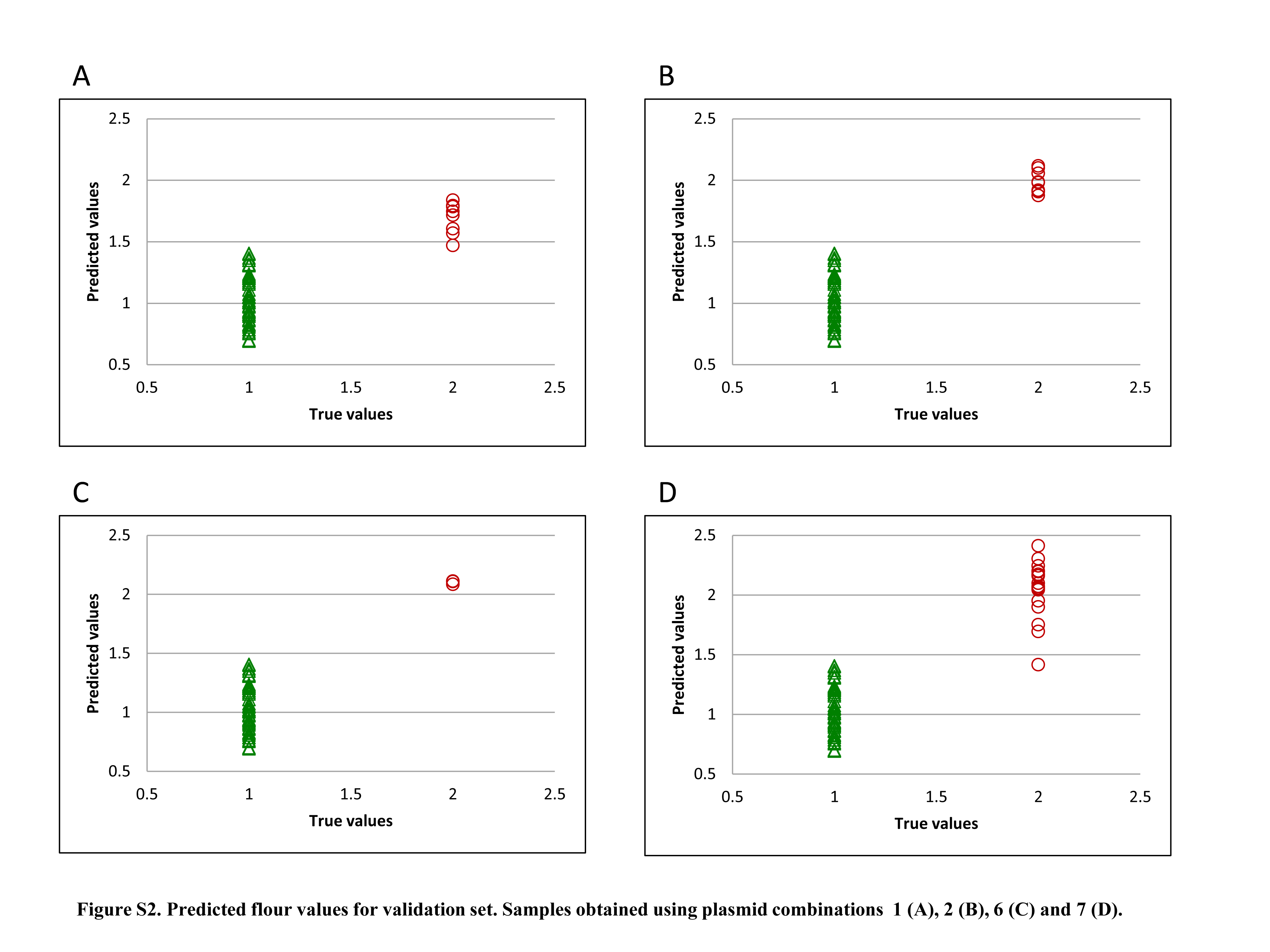

Supplement: S2 Fig — Samples obtained using plasmid combinations 1(A), 2 (B), 6 (C) and 7 (D). (TIF) [file pone.0152292.s002.tif]
