# Supplementary material for: Effective Identification of Low-Gliadin Wheat Lines by Near Infrared Spectroscopy (NIRS): Implications for the Development and Analysis of Foodstuffs Suitable for Celiac Patients
Source: PLoS One. 2016 Mar 28;11(3):e0152292. doi: 10.1371/journal.pone.0152292 (PMC4809495; doi:10.1371/journal.pone.0152292)
Supplement: S1 Table — (DOCX) [file pone.0152292.s003.docx]

**S1 Table. Plasmid combinations used: Combination number 0 indicates BW208 wild type wheat and numbers 1 – 9 show the plasmid combinations used to generate lines in years 2010, 2011, 2012, 2013 and 2014.**

| **RNAi Combination** | **Plasmid 1** | **Plasmid 2** | **Number of lines** | | | | | **Total Samples** |
| --- | --- | --- | --- | --- | --- | --- | --- | --- |
|  |  |  | **2010** | **2011** | **2012** | **2013** | **2014** |  |
| 0 | NA | NA | 6 | 58 | 36 | 9 | 47 | 156 |
| 1 | NA | pDhp_α/βZR | 0 | 0 | 9 | 9 | 9 | 27 |
| 2 | NA | pDhp_ω8ZR | 0 | 0 | 12 | 12 | 9 | 33 |
| 3 | NA | pDhp_ω/α | 15 | 21 | 9 | 9 | 9 | 63 |
| 4 | NA | pGhp_ω/α | 9 | 12 | 9 | 9 | 77 | 116 |
| 5 | pDhpg8.1 | pDhp_ω/α | 22 | 30 | 12 | 12 | 12 | 88 |
| 6 | pDhp_α/βZR | pDhp_ω4ZR | 0 | 0 | 3 | 3 | 3 | 9 |
| 7 | pDhp_α/βZR | pDhp_ω8ZR | 0 | 0 | 15 | 15 | 18 | 48 |
| 8 | pDhp_ω/α | pGhp_ω/α | 6 | 8 | 0 | 0 | 0 | 14 |
| 9 | pGhpg8.1 | pGhp_ω/α | 6 | 10 | 0 | 0 | 0 | 16 |

**Figure S1.** Predicted flour values for training set. Samples obtained using Plasmid Combinations 1 (A), 2 (B), 6 (C), 7 (D), 8 (E) and 9 (F).

**Figure S2.** Predicted flour values for validation set. Samples obtained using Plasmid Combinations 1 (A), 2 (B), 6 (C), and 7 (D).
